# The Impact of COVID-19 Pandemic on Emergency Department Visits at a Canadian Academic Tertiary Care Center

**DOI:** 10.5811/westjem.2021.2.49626

**Published:** 2021-07-19

**Authors:** Edmund S.H. Kwok, Glenda Clapham, Samantha Calder-Sprackman

**Affiliations:** * University of Ottawa, Department of Emergency Medicine, Ottawa, Ontario, Canada; † Ottawa Hospital Research Institute, Ottawa, Ontario, Canada

## Abstract

**Introduction:**

Public health response to the coronavirus 2019 (COVID-19) pandemic has emphasized social distancing and stay-at-home policies. Reports of decreased emergency department (ED) visits in non-epicenters of the outbreak have raised concerns that patients with non-COVID-19 emergencies are delaying or avoiding seeking care. We evaluated the impact of the pandemic on ED visits at an academic tertiary care center.

**Methods:**

We conducted an observational health records review between January 1–April 22, 2020, comparing characteristics of all ED visits between pre- and post-pandemic declaration by the World Health Organization. Measures included triage acuity, presenting complaints, final diagnoses, disposition, and mortality. We further examined three time-sensitive final diagnoses: stroke; sepsis; and acute coronary syndrome (ACS).

**Results:**

In this analysis, we included 44,497 ED visits. Average daily ED visits declined from 458.1 to 289.0 patients/day (−36.9%). For the highest acuity triaged patients there was a drop of 1.1 patients/day (−24.9%). Daily ED visits related to respiratory complaints increased post-pandemic (+14.1%) while ED visits for many other complaints decreased, with the greatest decline in musculoskeletal (−52.5%) and trauma (−53.6%). On average there was a drop of 1.0 patient/day diagnosed with stroke (−17.6%); a drop of 1.6 patients/day diagnosed with ACS (−49.9%); and no change in patients diagnosed with sepsis (pre = 2.8 patients/day; post = 2.9 patients/day).

**Conclusion:**

Significant decline in ED visits was observed immediately following formal declaration of the COVID-19 pandemic, with potential for delayed/missed presentations of time-sensitive emergencies. Future research is needed to better examine long-term clinical outcomes of the decline in ED visits during pandemics.

## INTRODUCTION

On March 22, 2020, the World Health Organization (WHO) designated the outbreak of a novel coronavirus (SARS-CoV-2) first reported in January 2020 as an international pandemic causing coronavirus disease 2019 (COVID-19).^1^–^3^ COVID-19 was thought to spread from person-to-person by respiratory droplets and contaminated surfaces or fomites, with asymptomatic transmission suspected.^4^–^6^ In an effort to “flatten the curve” public health response to COVID-19 encouraged social distancing, self-isolation, and stay-at-home policies, employing media campaigns that highlighted the experiences in Lombardy, Italy, and New York City, NY, where hospitals were overwhelmed by COVID-19.^7^

Shortly after the WHO’s pandemic declaration, anecdotal reports of emergency department (ED) visits plummeting occurred in many cities that were not overwhelmed by COVID-19 outbreaks. At our own tertiary care hospital in Canada’s capital, Ottawa, we observed daily ED visits drop to as low as ~50% compared to the same time period the prior year. At our center, confirmed COVID-19 admissions were limited (as of April 22, 2020, Ottawa had eight COVID-19 patients in intensive care, and 22 COVID-19 patients on inpatient wards^8^) and had not overwhelmed acute hospital capacity. The sudden drop in ED visits caused concern that patients with non-COVID-19 emergencies were delaying or avoiding seeking appropriate ED care during this pandemic.

We sought to rapidly review the immediate impact of the COVID-19 pandemic on ED visits at a tertiary care hospital not overwhelmed with COVID-19 admissions. We aimed to characterize and compare trends of pre- vs post-COVID-19 ED populations in terms of the Canadian Triage Acuity Score (CTAS) level, presenting complaints, discharge/admission diagnoses, and patient flow metrics. In addition, we sought to examine the effect of the pandemic on ED visits and mortality rates of three time-sensitive diagnoses: stroke; sepsis; and acute coronary syndrome (ACS).

## METHODS

### Design

We conducted a retrospective observational electronic health records (EHR) review.

### Setting

The Ottawa Hospital (TOH) is a 1202-bed academic tertiary care hospital with the ED receiving >174,000 visits per year. It is the main regional referral center for specialized services including trauma, stroke, neurosurgical, thoracic, oncological, and vascular emergencies. Adjacent to TOH is the regional cardiac center, the Ottawa Heart Institute, which receives prehospital Code STEMI (ST-elevation myocardial infarct) cases bypassing TOH EDs. It was not included in this study.

### Patient Population and Time Period

We included all patients presenting to TOH ED between January 1, 2019–April 22, 2020. We excluded all patients who were “direct-to-service,” which included patients already assessed at another hospital/outpatient clinic being transferred directly for admission to a specialized service at TOH. We used the date March 11, 2020, when the WHO declared COVID-19 to be an official pandemic, to define pre- and post-pandemic periods.

### Measures

We collected ED visit characteristics including patient demographics, presenting complaints, final diagnoses, and disposition. Mortality rates were observed for the entirety of patients’ ED or in-patient stays. We also collected data on patients’ CTAS, which is a triage tool used internationally to allow EDs and their staffs to prioritize patient care requirements upon arrival to the ED. Levels of CTAS range from 1 (most acute) to 5 (least acute).^9^

For presenting complaints and final diagnoses, two authors independently reviewed all primary chief complaints listed for each ED visit, as well as final discharge/admission diagnoses, and assigned them into the most appropriate categories based on symptom- or specialty-related headings. Any discrepancies were resolved with discussion between the reviewers, with arbitration by the third author if necessary. We used a similar process to critically review all discharge/admission diagnoses for three time-sensitive emergencies: stroke; sepsis; and ACS.

**Population Health Research Capsule**

What do we already know about this issue?

*Responses to the coronavirus disease 2019 (COVID-19) pandemic have emphasized social distancing and stay-at-home policies with subsequent reports of decreased emergency department (ED) visits*.

What was the research question?

*We evaluated the impact of the pandemic on ED visits at a center not overwhelmed with COVID-19 admissions*.

What was the major finding of the study?

*Decline in ED visits including time-sensitive emergencies was observed after declaration of a pandemic*.

How does this improve population health?

*Public health responses to pandemics affect ED visit behaviors. Further research is needed to examine long-term clinical outcomes of the decline in ED visits*.

### Data Collection

The Ottawa Hospital transitioned to Epic EHR (Epic Systems Corporation, Verona, WI) in June 2019. A quality improvement coordinator with Epic-reporting expertise pulled the required data elements from the EHR using integrated reporting functionalities and entered the data into a Microsoft Excel database (Microsoft Corporation, Redmond, WA) for further analysis. We retrieved historical patient volume data from TOH’s previous performance-measurement data warehouse.

### Data Analysis

We present patient demographics, CTAS acuity, presenting complaints, final diagnoses, process measures, time metrics, and mortality using descriptive statistics. For comparison between pre- and post-pandemic periods, we examined the total number of ED visits within each time period, as well as the number of ED visits per day. We plotted relevant results temporally to provide visual trends over time, with annotation to provide context around specific milestones. We assumed normal distributions and performed statistical analysis using Student’s two-sided t-test to compare pre- vs post-pandemic periods, and chi-squared test for comparison of proportions, with *P*-value of <0.05 considered to be significant.

### Ethical Considerations

We obtained research ethics approval for this project by the Ottawa Hospital Research Institute Research Ethics Board, dated Apr 24, 2020, protocol ID# 20200262-01H.

## RESULTS

A total of 44,497 ED visits met our inclusion/exclusion criteria during the study period (32,068 in pre-pandemic; 12,429 in post-pandemic) (Table 1). The mean age was 49.9 years old with 46.5% being male patients. Overall, average daily ED visits declined from 458.1 patients/day in the pre-pandemic period, to 289.0 patients/day in the post-pandemic period (−36.9%). There was a significant decrease in the proportion of patients with incomplete ED visits (ie, leaving without being seen, etc), from 8.6% in the pre-pandemic period to only 3.5% in the post-pandemic period.

**Figure 1:**
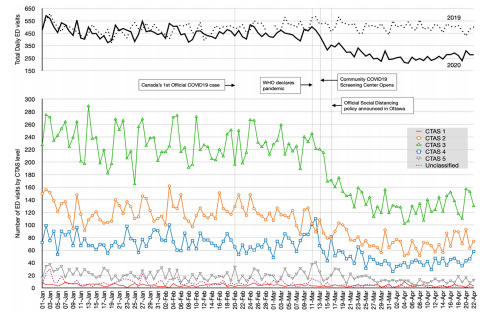
Number of emergency department (ED) visits according to triage CTAS level over time. The floating graph summarizes total daily ED visits for the study year (2020) compared to historical volumes from previous year (2019). *ED*, emergency department; *CTAS*, Canadian Triage Acuity Scale.

Relative CTAS levels distribution remained stable throughout the study period, with the exception of an increase in the proportion of CTAS 5 patients (pre: 4.5%, post: 5.0%). For the most severe and critical CTAS 1 acuity patients, on average there was a significant drop of 1.1 patients/day (−24.9%) in the post-pandemic period. For the second most critical CTAS 2 acuity patients, on average there was a significant drop of 45.9 patients/day (−37.7%). There was a sharp drop in overall ED visits immediately following the WHO declaration of a pandemic, followed by a second acute sustained drop in ED visits immediately after the city’s local announcement of social distancing policies (Figure 1).

The distribution of chief complaints presenting to the ED remained similar between the pre-/post-pandemic periods except for a number of categories (Table 1). The only categories that increased in proportion relative to all presenting complaints were *respiratory* (pre: 10.3%, post: 18.5%), *mental health* (pre: 5.8%, post: 6.3%), and *vascular* (pre: 0.1%, post: 0.2%). The top five presenting complaint categories with the greatest absolute numbers of decline in average daily ED visits were the following: 1) *trauma/environmental* with a drop of 32.2 patients/day (−53.6%); 2) *abdominal pain/gastrointestinal (GI)* with a drop of 30.0 patients/day (−42.3%); 3) *musculoskeletal* with a drop of 25.5 patients/day (−52.5%); 4) *other* with a drop of 23.3 patients/day (−40.2%); and 5) *cardiac* with a drop of 20.6 patients/day (−37.6%).

**Figure 2:**
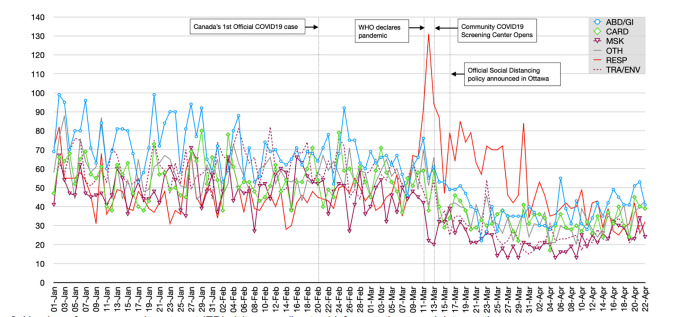
Number of emergency department (ED) visits according to chief presenting complaint over time. The bold red line represents the only chief complaint (respiratory) that increased in the post-pandemic period. [The other lines represent the top five chief complaints that demonstrated the greatest drop in absolute average number of daily ED visits in the post-pandemic period.] *ABD/GI*, abdominal pain/gastrointestinal; *CARD*, cardiac; *MSK*, musculoskeletal; *NEURO*, neurological; *OTH*, other; *RESP*, respiratory; *TRA/ENV*, trauma/environmental.

There was a volume decline in all presenting complaint categories except for *respiratory* complaints, which rose acutely following the WHO declaration of the COVID-19 pandemic (Figure 2). At its peak on March 12, 2020, there were 131 ED visits related to *respiratory* complaints (27.6% of all ED visits) that day. There was a subsequent drop in patients presenting with *respiratory* complaints two days later, coinciding with the opening of Ottawa’s first community COVID-19 screening center. By the end of March, all complaints had sustained decline in volume compared to pre-pandemic levels.

The distribution of final diagnoses also changed following the WHO pandemic declaration. Diagnoses related to *respiratory complaints* increased from 4.2% to 6.1% of all diagnoses; *infectious* increased from 13.2% to 20.0%; and *mental health* increased from 4.6% to 5.5%. The top five final diagnosis categories with the greatest absolute numbers of decline in average daily ED visits were the following: 1) *musculoskeletal* with a drop of 48.8 patients/day (−52.9%); 2) *abdominal pain/GI* with a drop of 21.4 patients/day (−38.4%); 3) *neurological* with a drop of 14.6 patients/day (−40.7%); 4) *cardiac* with a drop of 14.5 patients/day (−33.3%); and 5) *other* with a drop of 9.4 patients/day (−29.4%).

**Figure 3:**
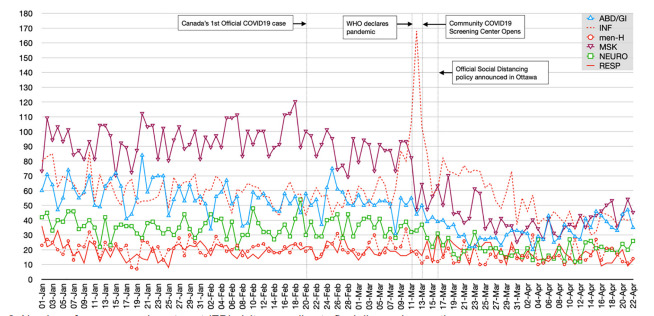
Number of emergency department (ED) visits according to final diagnosis over time. The red lines (bold, dashed, and dotted) represent the final diagnosis categories that experienced an increase in the post-pandemic period. The other colored lines represent the top three final diagnosis categories that experienced the greatest drop in absolute average number of daily ED visits in the post-pandemic period. *ABD/GI*, abdominal pain/gastrointestinal; *INF*, infectious; *men-H*, mental health; *MSK*, musculoskeletal; *NEURO*, neurological; *RESP*, respiratory.

Patients diagnosed with *infection*-related issues spiked immediately after WHO’s declaration of the COVID-19 pandemic, peaking at 168 ED visits (35.4%) on March 12, 2020 (Figure 3). The number of patients diagnosed with *mental health* and *respiratory*-related issues appeared to be stable over time. Diagnoses related to *musculoskeletal*, *abdominal/GI*, and *neurological* issues had sustained declines in the post-pandemic study period.

There was a significant increase in overall mortality rate for all ED visits in the post-pandemic period (pre: 1.1%, post: 1.6%), but no difference in mortality within the three subgroups of *stroke, ACS*, and *sepsis* (Table 2). There was a significant drop in average daily ED visits for *stroke* (5.8 patients/day in pre-pandemic; 4.8 patients/day in post-pandemic) and *ACS* (3.3 patients/day in pre-pandemic; 1.7 patients/day in post-pandemic), but no significant change in average daily number of ED patient diagnoses with *sepsis* (2.8 patients/day in pre-pandemic; 2.9 patients/day in post-pandemic).

Patient flow metrics significantly improved in the post-pandemic period. Physician initial assessment, defined as time from patient arrival to the ED to the time when first seen by a physician, improved by one hour (hr) and 50 minutes (min) (pre: 3hr 00 min, post: 1hr 10 min). Average ED length of stay for both discharged and admitted patients also significantly improved by 2 hr 12 min, and 11 hr 35 min, respectively. Finally, average total hospital length of stay for admitted patients decreased by 21 hr 39 min (pre: 214 hr 35 min, post: 192 hr 54 min).

## DISCUSSION

Following WHO’s declaration of COVID-19 as an official pandemic, we found a significant drop in overall visits to our ED. Patients presenting to the ED with respiratory and infectious issues sharply increased, while visits related to many other complaints decreased. Musculoskeletal- and trauma-related complaints appear to be the most impacted; this may in part have been due to social distancing and stay-at-home public health messaging resulting in fewer outdoor activities and vehicles on the road. It is important to note the drop in absolute numbers of patients who presented to the ED with potentially life-threatening CTAS 1 and 2 acuities (−47 patients/day; a 37.3% decline), strokes (−1.0 patient/day; a 17.6% decline), and myocardial infarction (MI) (−1.6 patients/day; a 49.9% decline). This a concerning proportion of patients with time-sensitive emergencies who were not presenting to the ED immediately following the pandemic declaration, given that there are no known physiological reasons for the prevalence of these conditions to be lower.

Interestingly, the number of patients diagnosed with sepsis appears to have remained stable, which may reflect the fact that septic patients often present to the ED via prehospital emergency medical services (EMS), and thus may be less affected by an individual’s fear of coming to the ED.^10^,^11^ Among patient groups whose volume of ED visits did not appear to be affected by the pandemic were those presenting with mental health-related issues. Anecdotally, physicians in our group reported seeing escalating cases of anxiety-related cases due to the COVID-19 pandemic; this may have been further augmented by closure of regular mental health community supports. Finally, we noticed significant improvements in all ED crowding and flow metrics. This is likely a result of the drop in hospital occupancy and improved internal operations after non-essential healthcare services were ceased during the pandemic period.

A decline in the number of non-COVID-19 patients presenting for emergency care has been anecdotally observed elsewhere, with numerous news media articles citing concerns of unintended consequences in North America.^12^,^13^ A regional hospital in Germany reported total ED visits to their center dropped by 23% within four weeks of admitting their first COVD-19 patient.^14^ Although the article did not report details on acuity levels, presenting complaints, or clinical outcomes, it did note a respective 53% and 30% decline in the hospital’s cardiology- and neurology-related ED populations. The authors postulated that these unintended consequences may have been a result of individuals’ extreme reactions to *dread risks*, defined as “low-probability events in which many people are killed at the same time,” such as the COVID-19 pandemic. Wong et al described a similar drop in overall ED visits in a community hospital in California, and interviews with patients confirmed *fear* as the overarching theme affecting decisions to avoid ED visits.^15^ There are few other studies examining the ED population as a whole, although more reports are being published with respect to how the COVID-19 pandemic may be affecting specific diagnoses such as acute MIs and strokes.^16^,^17^

Our findings also support the risk-avoidance behavior of ED patients with non-COVID-19 related issues in the setting of this pandemic. However, we did not power the study to robustly examine mortality rates for all subgroups of patients (due to limited time frame), and it is difficult to fully understand meaningful clinical impact. We did note an increased overall mortality rate in our study population, but this may simply be a reflection of the drop in non-emergent ED visits in the post-pandemic period rather than a true increase in severity of disease. Of note, our national statistics agency StatsCan found no increase in “excess deaths” between January 1–March 31, 2020 when compared to the same time period in the previous year.^18^ It is very difficult to accurately attribute any potential delayed/avoided ED visit directly to patients’ fears and behaviors in response to the pandemic. Future studies are needed to help identify this subgroup of patients who delayed ED presentation as a result of the pandemic, and to further examine relevant clinical consequences.

## LIMITATIONS

There are a number of important limitations to our study. Firstly, this was a single-center study in North America and may not reflect nuances around ED visit behaviors of patients in other healthcare systems. Although our center is the regional referral center for specialized emergencies including stroke code bypass, STEMI cases identified in the field by EMS are redirected to a separate cardiac center and thus were not included in this study. As a result, our findings may underestimate the potential impact on ED visits related to cardiac and ACS presentations noted in our findings. Secondly, our findings reflect a center with relatively low COVID-19 burden in terms of admissions and critical care resources, and thus should be interpreted in relation to similar centers that were not epicenters of the COVID-19 pandemic. Thirdly, the pre-/post-design was limited by our institution’s recent switch from paper charts to full Epic EHR; thus, we were unable to directly compare data from the same time period from previous year(s) without extensive manual chart review. However, we do not believe there are any seasonal variation factors between January-March vs March-April that would significantly invalidate our data. Finally, given the nature of a timely rapid review our study period was limited to just over a month past the WHO declaration of pandemic status. Future research with more detailed individual chart reviews are needed to assess delayed findings and clinical significance.

## CONCLUSION

Significant decline in ED visits was observed immediately following declaration of global pandemic status, with potential for delayed/missed presentations of time-sensitive emergencies. We believe it is important for public health communication strategies to take our findings into account, as messaging regarding staying at home may have created potential extreme reactions to dread risks. Future research is needed to examine long-term and impactful clinical outcomes related to significant decline in ED visits during pandemics.

## Figures and Tables

**Table 1:** Patient and emergency department visit characteristics between pre- and post-COVID-19 pandemic status.

	All Patients	Total # of ED visits			Average # of ED visits per day		
	
		Pre-pandemic	Post-pandemic	P-value	Pre-pandemic	Post-pandemic	P-value
Total ED Visits (N)	44,497	32,068	12,429		458.1	289.0	
Mean Age (yrs)	49.9	49.8	50.2	<0.05			
Gender, n(%)							
Male	20,678(46.5)	14,701(45.8)	59,77(48.1)	<0.05	210.0	139.0	<0.05
Female	23,761(53.5)	17,326(54.0)	64,35(51.8)	<0.05	247.5	149.7	<0.05
No gender documented	58(0.0)	41(0.0)	17(0.0)	1	0.6	0.4	0.22
CTAS acuity level, n(%)							
1	450(1.0)	308(1.0)	142(1.1)	0.35	4.4	3.3	<0.05
2	11,767(26.4)	8,513(26.5)	3,254(26.2)	0.52	121.6	75.7	<0.05
3	22,325(50.2)	16,112(50.2)	6,213(50.0)	0.71	230.2	144.5	<0.05
4	7,153(16.1)	5,103(15.9)	2,050(16.5)	0.12	72.9	47.7	<0.05
5	2,064(4.6)	1,445(4.5)	619(5.0)	<0.05	20.6	14.4	<0.05
No acuity documented	738(1.7)	587(1.8)	151(1.2)	<0.05	8.4	3.5	<0.05
Chief Presenting Complaint, n(%)							
Abdominal/Gastrointestinal	6,735(15.1)	4,972(15.5)	1,763(14.2)	<0.05	71.0	41.0	<0.05
Cardiac	5,315(11.9)	3,842(12.0)	1,473(11.9)	0.77	54.9	34.3	<0.05
Infectious	1,034(2.3)	725(2.3)	309(2.5)	0.21	10.4	7.2	<0.05
Mental Health	2,651(6.0)	1,865(5.8)	786(6.3)	<0.05	26.6	18.3	<0.05
Musculoskeletal	4,403(9.9)	3,408(10.6)	995(8.0)	<0.05	48.7	23.1	<0.05
Neurological	3,820(8.6)	2,772(8.6)	1,048(8.4)	0.50	39.6	24.4	<0.05
Obstetrical/Gynecological	885(2.0)	650(2.0)	235(1.9)	0.50	9.3	5.5	<0.05
Other	5,530(12.4)	4,045(12.6)	1,485(11.9)	<0.05	57.8	34.5	<0.05
Respiratory	5,593(12.6)	3,288(10.3)	2,305(18.5)	<0.05	47.0	53.6	<0.05
Trauma/Environmental	5,402(12.1)	4,204(13.1)	1,198(9.6)	<0.05	60.1	27.9	<0.05
Urological	1,153(2.6)	853(2.7)	300(2.4)	0.08	12.2	7.0	<0.05
Vascular	67(0.2)	45(0.1)	22(0.2)	<0.05	0.6	0.5	0.43
General Weakness/Medical	1,852(4.2)	1,359(4.2)	4,93(4.0)	0.34	19.4	11.5	<0.05
Undefined	57(0.1)	40(0.1)	17(0.1)	1	0.6	0.4	0.18
Final ED Discharge / Admission Diagnosis, n(%)							
Abdominal/Gastrointestinal	5,367(12.1)	3,894(12.1)	1,473(11.9)	0.56	55.6	34.3	<0.05
Cardiac	4,294(9.7)	3,047(9.5)	1,248(10.0)	0.11	43.5	29.0	<0.05
General Medical	1,563(3.5)	1,112(3.5)	451(3.6)	0.61	15.9	10.5	<0.05
Hematological	434(1.0)	337(1.1)	97(0.8)	<0.05	4.8	2.3	<0.05
Infectious	6,732(15.1)	4244(13.2)	2,488(20.0)	<0.05	60.6	57.9	<0.05
Mental Health	2,168(4.9)	1,489(4.6)	679(5.5)	<0.05	21.3	15.8	<0.05
Musculoskeletal	8,337(18.7)	6,465(20.2)	1,872(15.1)	<0.05	92.4	43.5	<0.05
Neurological	3,413(7.7)	2,502(7.8)	911(7.3)	0.08	35.7	21.2	<0.05
Obstetrical/Gynecological	1,086(2.4)	778(2.4)	308(2.5)	0.54	11.1	7.2	<0.05
Oncological	362(0.8)	264(0.8)	98(0.8)	1	3.8	2.3	<0.05
Other	3,225(7.2)	2,249(7.0)	976(7.9)	<0.05	32.1	22.7	<0.05
Respiratory	2,109(4.7)	1,357(4.2)	752(6.1)	<0.05	19.4	17.5	0.07
Toxicological	615(1.4)	404(1.3)	211(1.7)	<0.05	5.8	4.9	0.10
Urological	1,199(2.7)	874(2.7)	325(2.6)	0.56	12.5	7.6	<0.05
Vascular	183(0.4)	113(0.4)	70(0.6)	<0.05	1.6	1.6	0.96
Undefined	3,410 (7.7)	2,939(9.2)	471(3.8)	<0.05	42.0	11.0	<0.05
ED Disposition, n(%)							
Admission to hospital	7,186(16.1)	4,910(15.3)	2,276(18.3)	<0.05	70.1	52.9	<0.05
Discharge from ED	34,118(76.7)	24,398(76.1)	9,720(78.2)	<0.05	348.5	226.0	<0.05
Incomplete (LBT, LWBS, LAMA, eloped, etc)	3,193(7.2)	2,760(8.6)	433(3.5)	<0.05	39.4	10.0	<0.05
Time Metrics, hr min (mean)							
Physician initial assessment	2:31	3:10	1:10	<0.05			
ED length of stay for pts discharged from the ED	5:40	6:18	4:06	<0.05			
ED length of stay for pts admitted from the ED	19:04	22:44	11:09	<0.05			
Inpatient hospital length of stay	207:49						

*ED*, emergency department; *LBT*, left before triage; *LWBS*, left without being seen; *LAMA*, left against medical advice; *pts*, paients; *hr*, hours; *min*, minutes, CTAS, Canadian Triage Acuity Scale.

**Table 2:** Overall mortality rates and average mortality per day between pre- and post-COVID-19 pandemic status for patients diagnosed with stroke, acute coronary syndrome, and sepsis.

	All Patients n(%)	Total # of ED visits n(%)			Average # of ED visits per day		
	
		Pre-pandemic	Post-pandemic	P-value	Pre-pandemic	Post-pandemic	P-value
All Diagnoses	44,497(100)	32,068(100)	12,429(100)		458.1	289.0	<0.05
Overall Mortality	550(1.2)	354(1.1)	196(1.6)	<0.05	5.1	4.6	0.28
in ED	54(0.1)	37(0.1)	17(0.1)	1	0.5	0.4	0.32
in Hospital	496(1.1)	317(1.0)	179(1.4)	<0.05	4.5	4.2	0.40
Stroke	613(100)	407(100)	206(100)		5.8	4.8	<0.05
Overall Mortality	59(9.6)	38(9.3)	21(10.2)	0.72	0.5	0.5	0.69
in ED	5(0.8)	4(1.0)	1(0.5)	0.52	0.1	0.0	0.73
in Hospital	54(8.8)	34(8.4)	20(9.7)	0.28	0.5	0.5	0.64
ACS	306(100)	234(100)	72(100)		3.3	1.7	<0.05
Overall Mortality	39(22.5)	26(11.1)	13(18.1)	0.12	0.4	0.3	0.50
in ED	26(8.5)	17(7.3)	9(12.5)	0.17	0.2	0.2	0.71
in Hospital	13(4.2)	9(3.8)	4(5.6)	0.51	0.1	0.1	0.57
Sepsis	316(100)	193(100)	123(100)		2.8	2.9	0.76
Overall Mortality	36(11.4)	22(11.4)	14(11.4)	1	0.3	0.3	0.92
in ED	3(0.9)	2(1.0)	1(0.8)	0.86	0.0	0.0	0.87
in Hospital	33(10.4)	20(10.4)	13(10.6)	0.95	0.3	0.3	0.87

* “in ED,” mortalities within the emergency department; “in Hospital,” after admission into hospital.

*ACS*, acute coronary syndrome.
